# Onset of acid-neutralizing action of a calcium/magnesium carbonate-based antacid using an artificial stomach model: an in vitro evaluation

**DOI:** 10.1186/s12876-021-01687-8

**Published:** 2021-03-06

**Authors:** Maxim Voropaiev, Deborah Nock

**Affiliations:** 1 Bayer Consumer Care AG, Peter Merian Straße 84, 4002 Basel, Switzerland; 2 Medical WriteAway, 63 Willowcroft Way Cringleford, Norwich, NR4 7JJ UK

**Keywords:** Acid-neutralizing action, Antacid, Calcium carbonate, Magnesium carbonate, Pepsin activity

## Abstract

**Background:**

Calcium carbonate antacids are potent over-the-counter antacids, made more effective by adding magnesium carbonate (as in Rennie, Bayer). However, published studies on their onset of action are scarce. Therefore, we carried out an in vitro study comparing Rennie and placebo under simulated conditions of the human stomach (artificial stomach model) to reconfirm the onset of action of Rennie.

**Methods:**

The validated Simulator of the Human Intestinal Microbial Ecosystem apparatus (SHIME, ProDigest, Belgium) was used, comprising five reactors simulating different parts of the human gastrointestinal tract. Both Rennie and placebo were dosed at two tablets per incubation over six independent, 2-h stomach incubations each. Primary objectives: to evaluate the time required to achieve pH 3.0, 3.5, 4.0 and 4.5, as well as the maximum pH reached. Secondary objective: to evaluate pepsin activity over the entire 2-h gastric incubation.

**Results:**

After addition of Rennie, the gastric medium reached a pH of 3.0 within 40 s. The maximum pH of 5.24 was maintained for almost 10 min. In contrast, the maximum pH with placebo was 1.28 during the entire gastric simulation. Furthermore, Rennie strongly reduced the activity of mucosa-damaging pepsin during the period of increased pH. With placebo, the lower pH resulted in consistently high loads of digested peptides, reflecting the high cumulative and instantaneous pepsin activity.

**Conclusions:**

New data is a critical component in informed decision making. Our data confirm the high efficacy and fast onset of acid-neutralizing action of Rennie, which begins to work within seconds.

## Background

Antacids have been widely used for many years for the symptomatic treatment of gastroesophageal reflux disease and mild, non-ulcer dyspepsia [1]. Calcium carbonate-based antacids are one of the most potent over-the-counter antacids [2], known to have a rapid, long-lasting, and effective neutralizing action that is increased by the addition of magnesium carbonate, as in Rennie^®^ (Bayer). In the lower esophagus or in the stomach, calcium and magnesium carbonate react with the hydrochloric acid to form water and soluble mineral salts. However, although Rennie is a well-established antacid that has been on the market for over 85 years, published studies on the onset of action of calcium carbonate are scarce. The scientific data demonstrating its onset of action were generated some time ago and remain unpublished. Much has changed since then, and it is time to reconfirm the onset of action for Rennie.

**Fig. 1:**
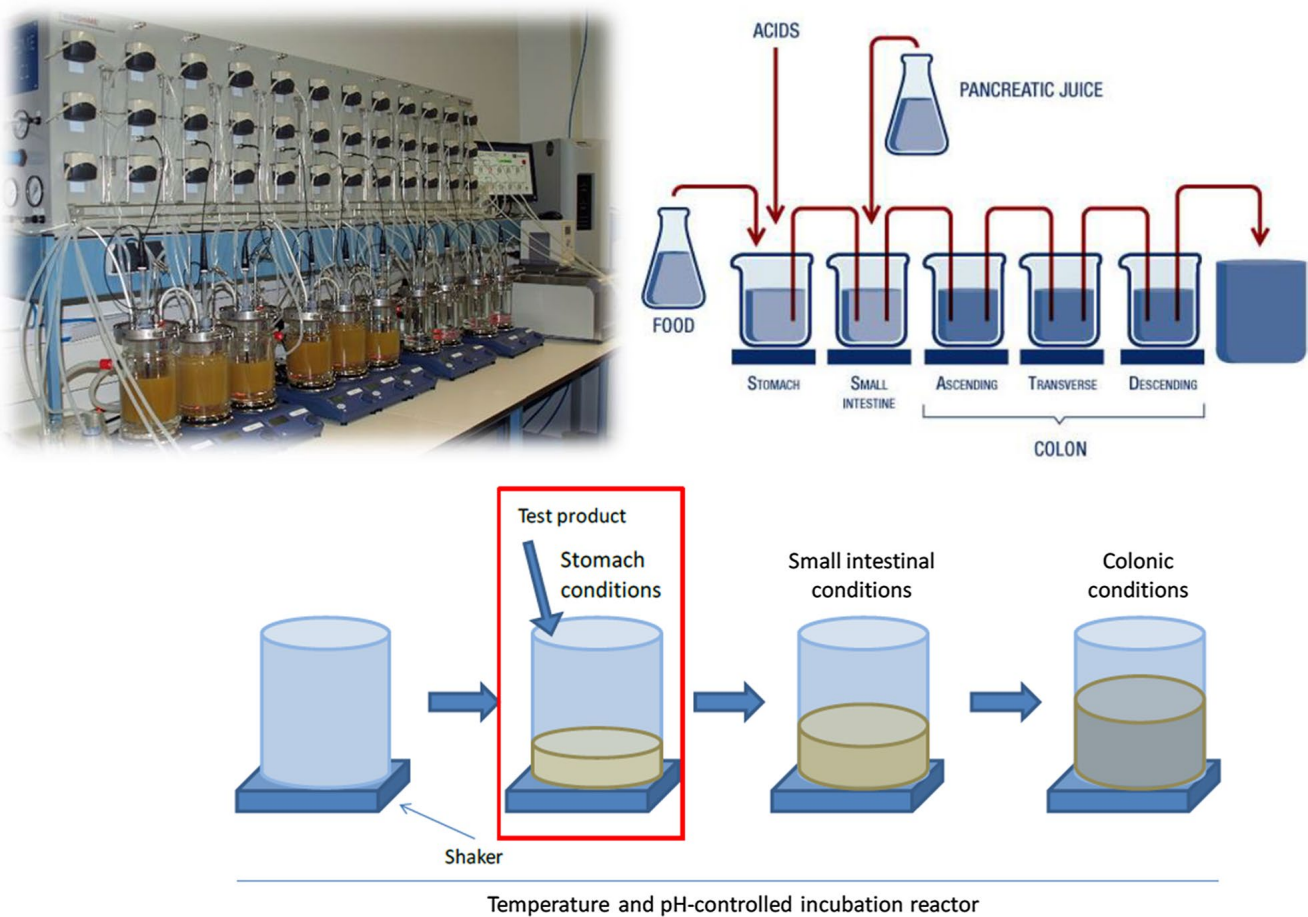
Overview of the SHIME (top) and adapted SHIME (bottom) apparatus. The adapted SHIME used one reactor in a sequential setup to simulate the stomach under fasted conditions (red rectangle). The SHIME apparatus (top right) represents the gastrointestinal tract of adult humans and incorporates peristaltic pumps (for automated administration of secretions, acid or base), pH controllers (to register online pH), heating elements (to maintain temperature at 37 °C), and magnetic stirrers (to homogenize contents). The first two reactors use the ‘fill-and-draw’ principle to simulate different steps in food uptake and digestion, with peristaltic pumps adding a defined amount of SHIME feed and gastric secretions (pepsin and HCl) to the stomach compartment and pancreatic/bile liquid to the small intestinal compartment. Peristaltic pumps also ensure emptying of the respective reactors after specified intervals. The last three compartments—continuously stirred reactors with constant volume and pH control—simulate the ascending, transverse, and descending colon. Retention time and pH of the different vessels are chosen to resemble in vivo conditions in the different parts of the gastrointestinal tract. The TWINSHIME setup (top left) consists of two SHIME systems in parallel. SHIME, Simulator of the Human Intestinal Microbial Ecosystem (ProDigest, Belgium). Image provided courtesy of ProDigest

Therefore, we carried out an in vitro study comparing Rennie and placebo under simulated conditions of the human stomach. The SHIME^®^ apparatus (Simulator of the Human Intestinal Microbial Ecosystem, ProDigest, Belgium) was used, which mimics the physiological and microbiological conditions representative of the human gastrointestinal tract (GIT). SHIME has been in extensive use for more than 25 years and has been validated using in vivo parameters. The system comprises a succession of five reactors simulating different parts of the human GIT (Fig. 1) [3]. The first two reactors simulate different steps in food uptake and digestion (stomach, small intestine), while the last three compartments simulate the ascending, transverse, and descending colon.

Our results should be of interest and relevance to healthcare providers and consumers. New data is a critical component in informed decision making and should be reassuring to those who have been recommending Rennie or selecting it for daily use based on its high efficacy and fast onset of action.

## Methods

### Product assessed

The antacid tested was Rennie Spearmint Chewable tablets (Bayer) (sugar-free), containing calcium carbonate (680 mg) and magnesium carbonate (80 mg) as active ingredients. The antacid was tested against placebo, both dosed at two tablets per incubation over six independent, 2-h stomach incubations each.

### Outcomes assessed

The endpoints of the gastric incubations are outlined in Table 1. The primary objectives were to evaluate the time required to achieve a pH of 3.0, 3.5, 4.0 and 4.5, as well as the maximum pH and duration of maximum pH, when the antacid was added to the simulated gastric environment. A secondary objective was to evaluate the pepsin activity over the entire 2-h gastric incubation using two methods: (1) cumulative pepsin activity, assessed via protein degradation over time; (2) instantaneous pepsin activity, assessed using a mathematical model constructed after a pH-based pre-test.

**Table 1:** Gastric incubation endpoints after administration of calcium/magnesium carbonate-based antacid (maximum recommended single dose) or placebo

pH profile	Pepsin activity
pH measured every 15 s within the first 5 min; thereafter, every 5 min until the end of the 2-h incubation	1) Cumulative activity: indirect measurement of digested amount of reference protein (BSA) after 0, 2.5, 5, 10, 15, 30, 45, 60, 90, 120 min^a^
Time to reach pH 3.0, 3.5, 4.0, 4.5 Maximum pH and duration of highest pH value (-0.1 pH unit)	2) Instantaneous activity: mathematical model, where pepsin activity was measured every 0.25 pH units from pH 1–7, using the same measurement of digested BSA as in 1)^b^

BSA, bovine serum albumin; TCA, trichloroacetic acid

^a^Upon precipitating the intact fraction of the reference protein BSA in the samples collected from the incubations using TCA, the digested protein fraction in the resulting supernatant was quantified via an absorbance measurement at 280 nm. This method required a pre-test to determine whether the antacid also resulted in absorbance at 280 nm and thus should be considered. Results of the pre-test indicated that the antacid did not interfere with the detection method at 280 nm; therefore, inclusion of additional blank incubations without BSA but with addition of the antacid was not required in the final experimental setup. ^b^Upon exposing BSA during 15 min to standardized pepsin levels, an absorbance measurement at 280 nm was performed after precipitating intact proteins in the sample via TCA. Thus, the digested protein fraction could be determined at each pH, allowing construction of a mathematical model of the relationship between pH and pepsin activity (as measured via the protein digestion method). Construction of the mathematical model led to a regression curve with strong correlation (R2 > 0.98), allowing accurate calculation of the instantaneous pepsin activity

To this purpose, we employed a standardized protocol using an adapted SHIME system that allows the conditions within one reactor to be modified to focus on one or more gastrointestinal region of interest (Fig. 1). By focusing on just one gastrointestinal compartment, multiple replicates could be run in parallel. In our case, the focus was on simulation of the stomach under fasted conditions. Protocols based on the InfoGest consensus method [4], and a standardized protocol from literature to evaluate acid-neutralizing profiles of actives [5] were used to ensure that simulation of the upper GIT was performed under the most representative conditions.

Briefly, a number of mucin-covered microcosms (simulating the mucus layer of the intestinal surface) with 100 mL of 0.1 N HCl (= total of 10 mmol H + , pH 1.0) were added to each stomach compartment containing relevant gastric compounds (mucins and salts such as KCl and NaCl) and the reference protein BSA (bovine serum albumin). At the start of the 2-h gastric incubation, the antacid and placebo were administered at a maximum single recommended dose. Thereafter, gastric acid secretion was simulated by pumping 0.1 N HCl into the stomach at a rate of 3 mL/min while gastric emptying was simulated by emptying the stomach at 1.5 mL/min, representing a slower emptying relative to the acid secretion rate. The content of each reactor was mixed continuously by means of magnetic stirring.

## Results

### Acid neutralization effects

**Fig. 2:**
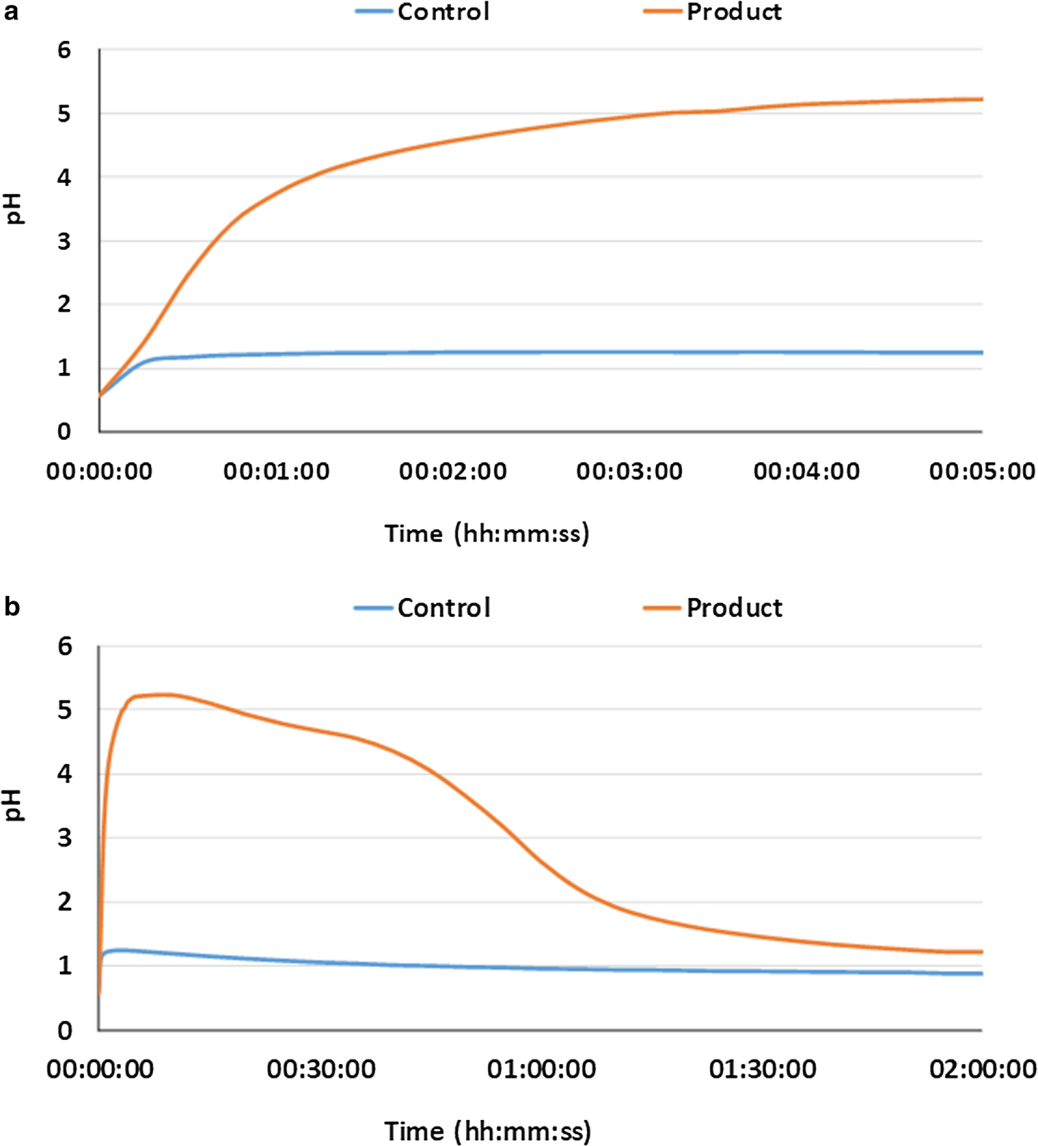
Average gastric pH profile after adding calcium/magnesium carbonate-based antacid or placebo. Average pH profile of six independent repetitions of gastric incubations after adding the calcium/magnesium carbonate-based antacid (Rennie Spearmint, Bayer) or placebo to the gastric medium. **a** First 5 min of incubation; **b** changes throughout the entire incubation period (2 h). Throughout the entire incubation, 0.1 N HCl was supplied at 3 mL/min, while gastric emptying was mimicked by removing the content at 1.5 mL/min

The pH of the gastric environment began at 1.0 and increased with addition of both antacid and placebo (Fig. 2). With antacid, the pH increased to > 3.0 within 40 s, and to > 4.5 at 1 min 54 s (Table 2). Overall, a pH > 3.0 was maintained for 56 min 1 s ± 1 min 9 s. The maximum pH of 5.24 was reached within 10 min and maintained for 9 min 56 s. Between 30 min and 1 h, the pH began to gradually decline due to the continuous influx of HCl.

**Table 2:** Acid-neutralizing activity of the calcium/magnesium carbonate-based antacid versus placebo (n = 6)

pH parameter	Placebo	Antacid
Time to reach pH:		
3.0	–	00:00:40 ± 00:00:02
3.5	–	00:00:53 ± 00:00:02
4.0	–	00:01:13 ± 00:00:04
4.5	–	00:01:54 ± 00:00:12
Maximum pH	1.28 ± 0.10	5.24 ± 0.07
Duration of maximum pH	00:13:05 ± 00:07:20	00:09:56 ± 00:00:44

Results presented as average ± standard deviation (units of time: hours:minutes:seconds)

In contrast, the pH did not reach pH 3.0 during the entire gastric simulation with placebo (max. pH 1.28).

### Pepsin activity

**Fig. 3:**
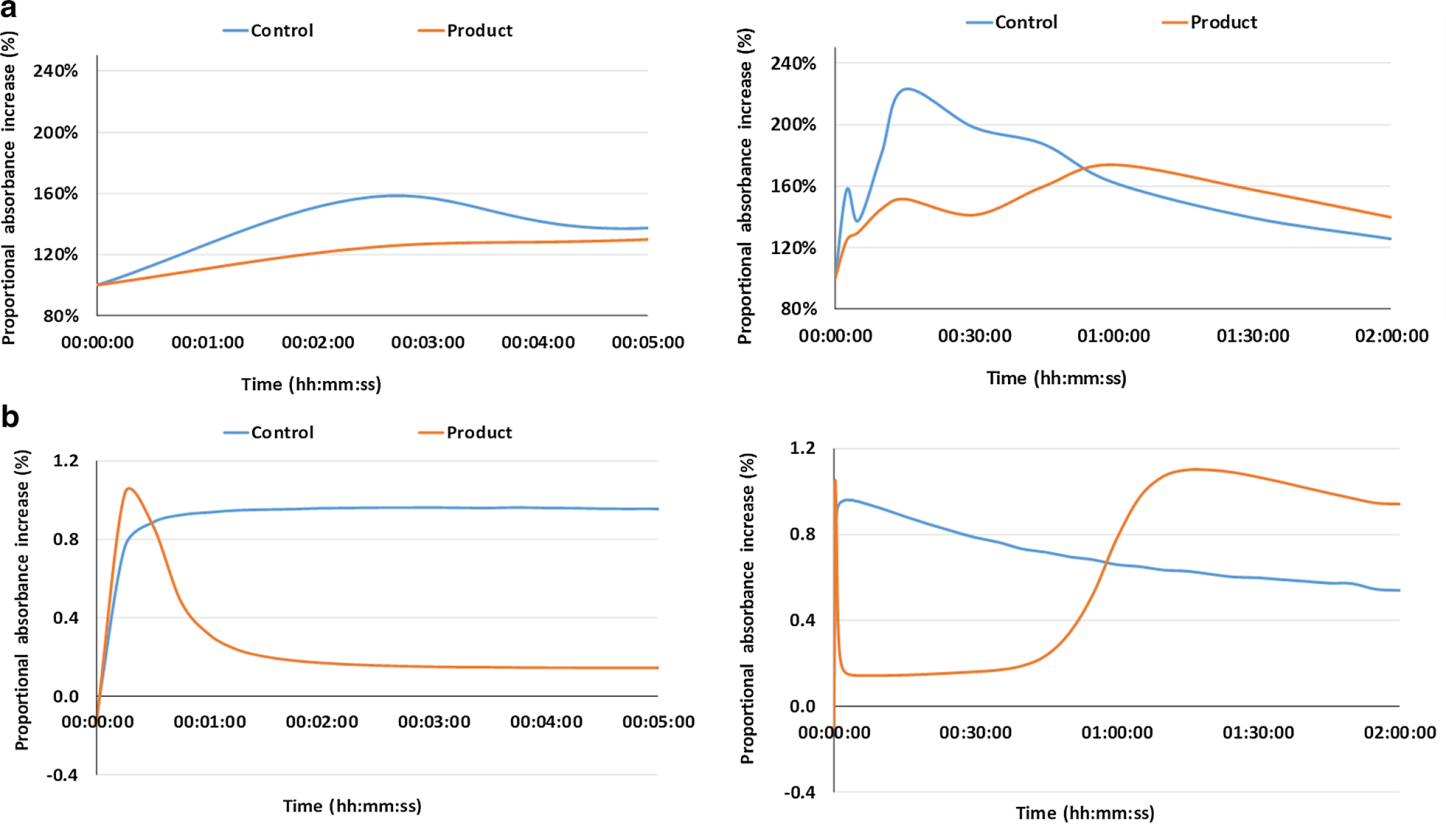
Average **a** cumulative and **b** instantaneous increase in gastric pepsin activity with antacid or placebo. The graphs reflect the concentration of digested peptide/protein fractions due to the action of pepsin on a reference protein (bovine serum albumin) during antacid or placebo incubations (n = 6) (absorbance values at 280 nm). Graphs on the left focus on the first 5 min of incubation, while those on the right show the changes throughout the entire incubation period

With the antacid, there was a decrease in both cumulative and instantaneous pepsin activity (Fig. 3) that was in line with the increase in pH values within the first 5 min and over the longer term (2 h). With placebo, the lower pH resulted in consistently high loads of digested peptides, reflecting the high cumulative and instantaneous pepsin activity (maximal peptide level between 15 and 30 min of incubation).

After 30 min (cumulative activity) and 45 min (instantaneous activity) of incubation, the declining pH values with antacid resulted in increased digested peptide fractions and thus increased pepsin activity, with maximum pepsin activity reached after 1 h of incubation. It should be noted that after 1 h of incubation, pepsin activity was higher for the antacid than placebo because the pH of the antacid incubations was closer to the optimum pH of pepsin activity (around 1.75). Due to the constant influx of gastric secretions at pH = 1.0, the pH in the placebo incubation had already decreased to below this optimum value.

## Discussion

Antacids are widely used in the treatment of mild-to-moderate symptoms of gastroesophageal disease such as heartburn, which occurs when acid travels up from the stomach to cause a burning sensation in the throat. Calcium carbonate has been used for decades to neutralize the acid and provide relief from heartburn [2]. However, published data for the onset of action of the calcium/magnesium carbonate-based antacid Rennie is lacking, and needed to be addressed to support confidence in its use.

Our in vitro study used a validated stomach model that confirmed the antacid has fast-acting and strong acid-neutralizing properties—a meaningful increase in pH was rapidly achieved within 40 s and maintained for almost one hour. This data is novel, as it was previously understood that the acid neutralization provided by Rennie began after 2 min; in fact, Rennie begins to work much faster than that, within seconds.

Pepsin is required to initiate the digestion of proteins, and a pH 1–2 is required for its activity; activity is limited at around pH 3.5–5 [6]. Gastric juices normally protect the gastric mucosa, but it may be susceptible to digestion at low pH values. Thus, the enzyme is also a known mucosa-damaging factor in heartburn and regurgitation, and its activity should be limited. After administration of Rennie in our study, the activity of pepsin was reduced both within the first 5 min and over the longer term (2 h). In vitro assessment has indicated that both calcium carbonate and magnesium carbonate have high anti-peptic activity (82% and 82%, respectively) [7].

Current technological advancements have allowed our experiment to be performed in the least intrusive manner. Repeating a clinical study seemed ethically unjustified, since new data would not be generated. Yet a simple in vitro acid neutralization test would not be relevant from a clinical practice perspective. We believe that a validated artificial stomach model was the best choice and a strength of the study, offering scientifically rigorous methodology in an environment closely mimicking human physiology. Future experiments comparing Rennie against other common antacids may be helpful to confirm its fast onset of acid neutralization.

## Conclusion

The calcium/magnesium carbonate-based antacid Rennie has strong acid-neutralizing effects. In this artificial stomach model, which mimics the physiological and microbiological conditions of the human GIT, the gastric medium reached a pH of 3.0 within 40 s and was maintained for almost 1 h. The maximum pH of 5.24 was maintained for almost 10 min. Furthermore, Rennie strongly reduced the activity of mucosa-damaging pepsin during the period of increased pH. These results contribute to the much-needed published literature regarding the onset of action of this well-established antacid.

## Data Availability

The datasets used and/or analyzed during the current study are available from the corresponding authors on reasonable request.

## Abbreviations

BSA: Bovine serum albumin
GIT: Gastrointestinal tract
SHIME: Simulator of the Human Intestinal Microbial Ecosystem
TCA: Trichloroacetic acid
